# Day-night rhythm of skeletal muscle metabolism is disturbed in older, metabolically compromised individuals

**DOI:** 10.1016/j.molmet.2020.101050

**Published:** 2020-07-11

**Authors:** Jakob Wefers, Niels J. Connell, Ciarán E. Fealy, Charlotte Andriessen, Vera de Wit, Dirk van Moorsel, Esther Moonen-Kornips, Johanna A. Jörgensen, Matthijs K.C. Hesselink, Bas Havekes, Joris Hoeks, Patrick Schrauwen

**Affiliations:** 1 Department of Nutrition and Movement Sciences, NUTRIM School for Nutrition and Translational Research in Metabolism, Maastricht University Medical Center, 6200 MD Maastricht, the Netherlands; 2 Division of Endocrinology, Department of Internal Medicine, Maastricht University Medical Center, 6200 MD Maastricht, the Netherlands

**Keywords:** Mitochondria, Skeletal muscle, Day-night rhythm, Insulin resistance

## Abstract

**Objective:**

Skeletal muscle mitochondrial function and energy metabolism displays day-night rhythmicity in healthy, young individuals. Twenty-four-hour rhythmicity of metabolism has been implicated in the etiology of age-related metabolic disorders. Whether day-night rhythmicity in skeletal muscle mitochondrial function and energy metabolism is altered in older, metabolically comprised humans remains unknown.

**Methods:**

Twelve male overweight volunteers with impaired glucose tolerance and insulin sensitivity stayed in a metabolic research unit for 2 days under free living conditions with regular meals. Indirect calorimetry was performed at 5 time points (8 AM, 1 PM, 6 PM, 11 PM, 4 AM), followed by a muscle biopsy. Mitochondrial oxidative capacity was measured in permeabilized muscle fibers using high-resolution respirometry.

**Results:**

Mitochondrial oxidative capacity did not display rhythmicity. The expression of circadian core clock genes *BMAL1* and *REV-ERBα* showed a clear day-night rhythm (p < 0.001), peaking at the end of the waking period. Remarkably, the repressor clock gene *PER2* did not show rhythmicity, whereas *PER1* and *PER3* were strongly rhythmic (p < 0.001). On the whole-body level, resting energy expenditure was highest in the late evening (p < 0.001). Respiratory exchange ratio did not decrease during the night, indicating metabolic inflexibility.

**Conclusions:**

Mitochondrial oxidative capacity does not show a day-night rhythm in older, overweight participants with impaired glucose tolerance and insulin sensitivity. In addition, gene expression of *PER2* in skeletal muscle indicates that rhythmicity of the negative feedback loop of the molecular clock is disturbed.

**ClinicalTrials.gov ID:**

NCT03733743.

## Highlights

• Diurnal rhythmicity of mitochondrial function is attenuated in older and metabolically compromised men.
• Rhythmicity of the biological clock gene PER2 is disturbed in skeletal muscle of these volunteers.
• Metabolic flexibility in 24-h metabolism is diminished, with a lack of a typical feeding-fasting cycle.

## 1 Introduction

Metabolic processes in the human body are highly dynamic and adapt rapidly to increased demand during physical activity and to energy intake in the postprandial period. The body's circadian timing system is intertwined with metabolism at various levels, thereby anticipating metabolic challenges, such as increased energy demand during the day. It is therefore not surprising that acute and chronic disturbance of the biological clock are related to disturbances in metabolic health. For example, chronic shift work is associated with increased risk for type 2 diabetes [1], and simulated shift work in human volunteers leads to glucose intolerance [2]. Furthermore, we have recently shown that a rapid day-night shift in healthy humans leads to a reduction in insulin sensitivity, which could specifically be attributed to a reduction in skeletal muscle insulin sensitivity [3].

In humans, circadian rhythmicity (i.e., 24-h rhythm that persists under constant conditions) or a 24-h day-night rhythm (i.e., recurring 24-h rhythm under free living conditions) has been observed for a variety of metabolic processes. One of the earliest reports observed higher glucose tolerance in the morning than in the afternoon [4]. Subsequent investigations showed rhythmicity in metabolic gene expression [5,6], protein abundance [7], and parts of the lipidome and metabolome [8]. We recently reported a pronounced day-night rhythm in human skeletal muscle mitochondrial oxidative capacity in young, lean, healthy volunteers [9]. Mitochondrial oxidative capacity was highest in the late evening, which was consistent with the pattern in whole-body energy expenditure. We also observed a day-night rhythm in the expression of the biological clock genes *BMAL1*, *PER2*, and *CRY1*, suggestive of metabolic and circadian cohesion in skeletal muscle.

Obesity, aging, and insulin resistance have been reported to result in both decreased mitochondrial oxidative capacity [10] and reduced metabolic flexibility [11]. Therefore, reduced mitochondrial function may mediate this metabolic inflexibility in skeletal muscle.

In light of these findings, it can be hypothesized that the day-night rhythm in skeletal muscle mitochondrial oxidative capacity we observed previously in young, healthy individuals is a prerequisite for metabolic health. However, so far human data about the day-night rhythmicity of skeletal muscle mitochondrial capacity and energy metabolism in older, metabolically comprised individuals is lacking. Here, we provide a detailed characterization of 24-h energy and substrate metabolism, including rhythmicity in skeletal muscle oxidative capacity using tissue from sequentially sampled skeletal muscle biopsies in older individuals with comprised metabolic health.

## 2 Materials and methods

### 2.1 Participants

Male, overweight volunteers between the ages of 40 and 75 years were recruited through advertisements in the vicinity of Maastricht. Participants were non-smokers and generally healthy, as determined by a medical questionnaire and examination by a physician. Only participants with a habitual bedtime of 11 PM ± 2 h and 7–9 h of sleep per day were included. Participants were excluded from the study if they performed shift work or had traveled across more than one time zone in the 3 months before the study. By means of the Morningness-Eveningness Questionnaire Self-Assessment Version 1.3 (MEQ-SA; Score between: 35–70), we excluded extreme morning or evening types. To establish volunteers with reduced metabolic health, we selected participants with impaired glucose tolerance and insulin sensitivity. To that end, participants had to fulfill at least one of 4 criteria in order to be included in the study: 1) impaired fasting glucose (IFG) 6.1 mmol/L-6.9 mmol/L; 2) impaired glucose tolerance (IGT) 7.8 mmol/L-11.1 mmol/L 2 h after 75 g glucose consumption; 3) HbA_1c_ of 5.7–6.4%; or 4) low insulin sensitivity defined as glucose clearance rate ≤ 360 ml/kg/min according to the Oral Glucose Insulin Sensitivity (OGIS) model [12]. Criteria 1 and 2 are derived from the World Health Organization recommendations [13], criteria 3 is derived from the definition of prediabetes from the American Diabetes Association [14]. The study was conducted in accordance with the principles of the declaration of Helsinki and approved by the Ethics Committee of the Maastricht University Medical Center. All participants provided written informed consent. The study was registered at https://clinicaltrials.gov with identifier NCT03733743.

### 2.2 Study conditions

During a 7-day run-in period prior to admission to the research facility, participants were instructed to adhere to a fixed lifestyle that resembled the experimental conditions during the overnight stay. During this run-in period, participants had to refrain from alcohol and caffeinated drinks, sleep only between 11 PM and 7 AM, and only eat 3 meals per day at 8 AM, 1 PM, and 6 PM. Standardized meals with fixed caloric content adjusted to the participants' needs (described below) were provided for the last 2 days before the visit to the lab. Furthermore, participants were instructed to refrain from exercise for the last 3 days of the run-in period. Adherence to the activity protocol and sleeping times at home was checked using actigraphy (Actiwatch) and a sleep diary. Mealtimes were registered in a food diary for 1 week, and meal contents were noted for the last 3 days before the visit to the lab.

#### 2.2.1 Overnight visit

All study procedures were performed as described previously in the study by van Moorsel et al. [9]. Participants were admitted to the research unit at 12 PM on test day 1 and stayed for 44 h in total under standardized conditions mimicking a real-life situation. The first test day was used to standardize and monitor meals, physical activity, and bedtime. Meals were provided at 1 PM and 6 PM in our research facilities. One hour after every meal, participants went for a 15-minute low-intensity walk accompanied by a researcher in order to standardize physical activity. Directly hereafter, participants were instructed to stand for 15 min before they were allowed to sit again. Between meals, physical activity, and tests, the participants stayed in a respiration chamber; a small room with a bed, toilet, sink, desk, chair, TV and computer. At 11 PM, the lights of the respiration chamber were turned off and the participants were instructed to try to sleep. During the first night, sleeping metabolic rate was measured by whole-room indirect calorimetry (Omnical, Maastricht Instruments, Maastricht, The Netherlands) [15].

On the second test day, participants were woken up at 6:30 AM and swallowed a telemetric pill for measurement of core body temperature (Equivital, Philips-Respironics, Murrysville PA, USA). Next, an intravenous cannula was placed in the forearm for subsequent blood-draws. The first blood-draw was at 8 AM, followed by an indirect calorimetry measurement using a ventilated hood while awake and at rest in supine posture to calculate resting energy expenditure and substrate oxidation. Directly hereafter, the first skeletal muscle biopsy was taken (described below). These measurements (blood draw, indirect calorimetry and skeletal muscle biopsy) were repeated 5 times within 24-h: at 8 AM, 1 PM, 6 PM, 11 PM, and 4 AM of the next day. Additional blood samples were taken at 2-h intervals (10 AM, 12 PM, 2 PM, 4 PM, 8 PM, 10 PM, 12 AM, 2 AM, 6 AM, and 8 AM). The meals on the second test day were provided immediately after the muscle biopsies and were thus delayed by approximately 1 h compared to the run-in period and the first test day. After the 11 PM biopsy, participants returned to the respiration chamber to sleep with lights off. At 4 AM, the participants were woken up, and the last measurements were performed, after which the subject was allowed to sleep until 7 AM. After the 8 AM blood draw, the study protocol ended. All indirect calorimetry measurements and muscle biopsies were performed in normal room light conditions.

#### 2.2.2 Study meals

Two days before admission to the metabolic research unit, and during the study, the participants were provided with standardized meals that were based on Dutch dietary guidelines and matched for energy expenditure. Caloric intake for consumption at home was calculated by multiplying the estimated resting metabolic rate, obtained with the Harris-Benedict formula [16] with an activity factor of 1.5. Participants were provided with optional extra snacks to eat with their meals if they were still hungry, up to an activity factor of 1.7. For the first test day in the laboratory, energy requirement was calculated by multiplying the estimated resting metabolic rate with an activity factor of 1.35, because of limited physical activity in the research facility. For the second test day, energy requirement was calculated by multiplying the sleeping metabolic rate of the first study night (measured by whole-room indirect-calorimetry) by 1.5.

During the study days, participants received 3 meals daily. Breakfast accounted for 21 energy% (E%), lunch for 30 E%, and dinner for 49 E%. Daily macronutrient composition was 52 E% as carbohydrates, 31 E% as fat (9 E% saturated), and 14 E% as protein. Breakfast and lunch were bread-based, and thus, the percentage of energy from carbohydrates was higher compared to the dinner. No snacks or drinks other than water were provided between meals.

### 2.3 Skeletal muscle biopsies

Five skeletal muscle biopsies were obtained from the vastus lateralis according to the Bergström method [17] under local anesthesia (1% lidocaine, without epinephrine). Each biopsy was taken from a separate incision at least 2 cm from the previous incision, moving from distal to proximal. The first biopsy was randomly taken from the left or right leg, and each subsequent biopsy was taken from the other leg. After the biopsies of 11 PM and 4 AM, participants lay in bed and tried to sleep. All biopsies were taken at least 4 h after the last meal to prevent any acute influence of meal ingestion. Part of the biopsy was immediately placed in ice-cold preservation medium (BIOPS, OROBOROS Instruments, Innsbruck, Austria) and used for the preparation of permeabilized muscle fibers and subsequent measurement of mitochondrial oxidative capacity. The remaining part of the muscle biopsy was immediately frozen in melting isopentane and stored at −80 °C until further analysis.

### 2.4 High-resolution respirometry

Permeabilized muscle fibers were prepared freshly from biopsies, as described previously [18]. In permeabilized muscle fibers, oxygen consumption was measured by high-resolution respirometry using an Oxygraph (OROBOROS Instruments). During the experiment, a multisubstrate-uncoupler protocol with malate, octanoylcarnitine, adenosine diphosphate (ADP), glutamate, succinate, and carbonylcyanide p-trifluoromethoxyphenylhydrazone (FCCP) was performed, as described previously [19]. The integrity of the outer mitochondrial membrane was assessed by the addition of cytochrome C upon maximal coupled respiration. All oxygen consumption measurements were performed in quadruplicate.

### 2.5 Indirect calorimetry

Oxygen consumption and carbon dioxide production were measured with an automated respiratory gas analyzer using a ventilated hood system (Omnical; IDEE, Maastricht, the Netherlands) and were used to calculate whole-body energy expenditure, respiratory exchange ratio (RER), and glucose and fat oxidation. Calculations of energy expenditure and substrate oxidation were made with the assumption of a negligible protein oxidation [20].

### 2.6 Core body temperature

After waking up on the second test day, participants ingested a thermometer capsule (Equivital, Philips-Respironics, Murrysville PA, USA) to measure core body temperature (CBT) for 24 h. Due to premature excretion of the capsule or technical failure, complete CBT measurements were obtained in only 11 participants.

### 2.7 Protein analysis

Western blot analyses were performed in Bioplex-lysates of human muscle tissue. Protein concentration was determined with the Bio-Rad RC/DC protein assay kit (Bio-Rad Laboratories, Veenendaal, The Netherlands). Equal amounts of protein were loaded on 12% TGX stain-free gels (Bio-Rad Laboratories) or 4–12% Bolt gradient gels (Novex, Thermo Fisher Scientific, Bleiswijk, The Netherlands). Proteins were transferred to nitrocellulose with the Trans-Blot Turbo transfer system (Bio-Rad Laboratories). Protein load was controlled in the stain-free gels [21] and blots and with the REVERT stain (REVERT total protein stain, LI-COR, Lincon, USA) in the 4–12% gradient gels. Primary antibodies: a cocktail of mouse monoclonal antibodies directed against human OXPHOS (dilution 1:5,000; ab110411, Abcam, Cambridge, UK), 2 mitochondrial markers directed against TOMM20 (dilution 1:10,000; ab186734; Abcam), porin/VDAC (dilution 1:5,000; sc-8828, Santa Cruz Biotechnology, Dallas, Texas), FIS-1 (dilution 1:1,000, sc-98900, Santa Cruz Biotechnology, Dallas, Texas), PINK-1 (dilution 1:2,000, sc-33796, Santa Cruz Biotechnology, Dallas, Texas), and OPA-1 (dilution 1:2500, 612,606, Becton Dickinson). The specific proteins were detected using secondary antibodies conjugated with IRDye680 or IRDye800 and were quantified with the CLx Odyssey Near Infrared Imager (Li-COR, Westburg, Leusden, The Netherlands).

### 2.8 Gene transcript quantification

RNA was isolated from 10 mg of muscle material by TRIzol lysis (Qiagen, Hilden, Germany). RNA was further purified by the RNeasy kit from Qiagen (Hilden, Germany). RNA yield was measured using a NanoDrop spectrophotometer (Thermo Fisher Scientific, Waltham, USA). The high-capacity RNA-to-cDNA kit from Applied Biosystems (Foster City, USA) was used for transcribing 200 ng of RNA to cDNA. Transcript abundance was determined using a CFX384 Real-Time System (Biorad-Laboratories, Veenendaal, NL). To minimize the variability in reference gene normalization, the geometric mean of three reference genes (RPL26, GUSB, and CYPB), which were individually stably expressed in time, was used. This geometric mean was used as the internal reference for comparative gene expression analysis in the remainder of the study [22].

### 2.9 Statistics

Data are presented as mean ± SEM (standard error of the mean) unless indicated otherwise. Statistical analyses were performed with the use of IBM Statistical Package for Social Sciences for MAC, version 23 (SPSS, Inc.). The effect of time on outcome variables was analyzed by repeated measures analysis of variance (ANOVA). In case of significance, Bonferroni adjusted post-hoc analyses were applied to look at differences between specific time-points. Statistical significance was defined as a p-value < 0.05. In addition, if repeated measures ANOVA resulted in a significant effect of time for targets of mRNA and protein expression and oxidative capacity states, we tested for rhythmicity using the JTK_Cycle package [23] in R 3.2.1. For this analysis, values were normalized to subject mean prior to analysis.

## 3 Results

### 3.1 Subject characteristics and circadian entrainment period

Twelve male volunteers with a mean age of 65 ± 9 years who were overweight (BMI: 30.3 ± 2.7 kg/m^2^) participated in the study. Subject characteristics are summarized in Table 1. A general overview of the study procedures is depicted in Figure S1. Core body temperature (CBT), which is under control of the central biological clock [24], was measured for 24 h beginning on day 2 and showed a typical day-night rhythm. The nadir occurred on average around 1 AM, indicating similar circadian entrainment of the participants (Figure S2).

**Table 1:** Descriptive characteristics during screening.

Parameter	
Age (years)	65 ± 9
Height (m)	1.78 ± 0.05
Body weight (kg)	96 ± 12
BMI (kg/m^2^)	30.3 ± 2.7
Body fat (%)	33 ± 4
Fasting plasma glucose (mmol/L)	5.7 ± 0.4
Fasting plasma insulin (μIU/mL)	13.8 ± 8.5
2-h plasma glucose (mmol/L)	7.3 ± 1.5
HbA_1c_ (%)	5.3 ± 0.5
Glucose clearance (ml/kg/min)	327 ± 38
MEQ-SA score	57 ± 9

Subject's characteristics at baseline. Values are depicted as mean ± SD (n = 12). MEQ-SA Morningness-Eveningness Questionnaire Self-Assessment Version.

### 3.2 Mitochondrial oxidative capacity lacks a day-night rhythm

**Figure 1:**
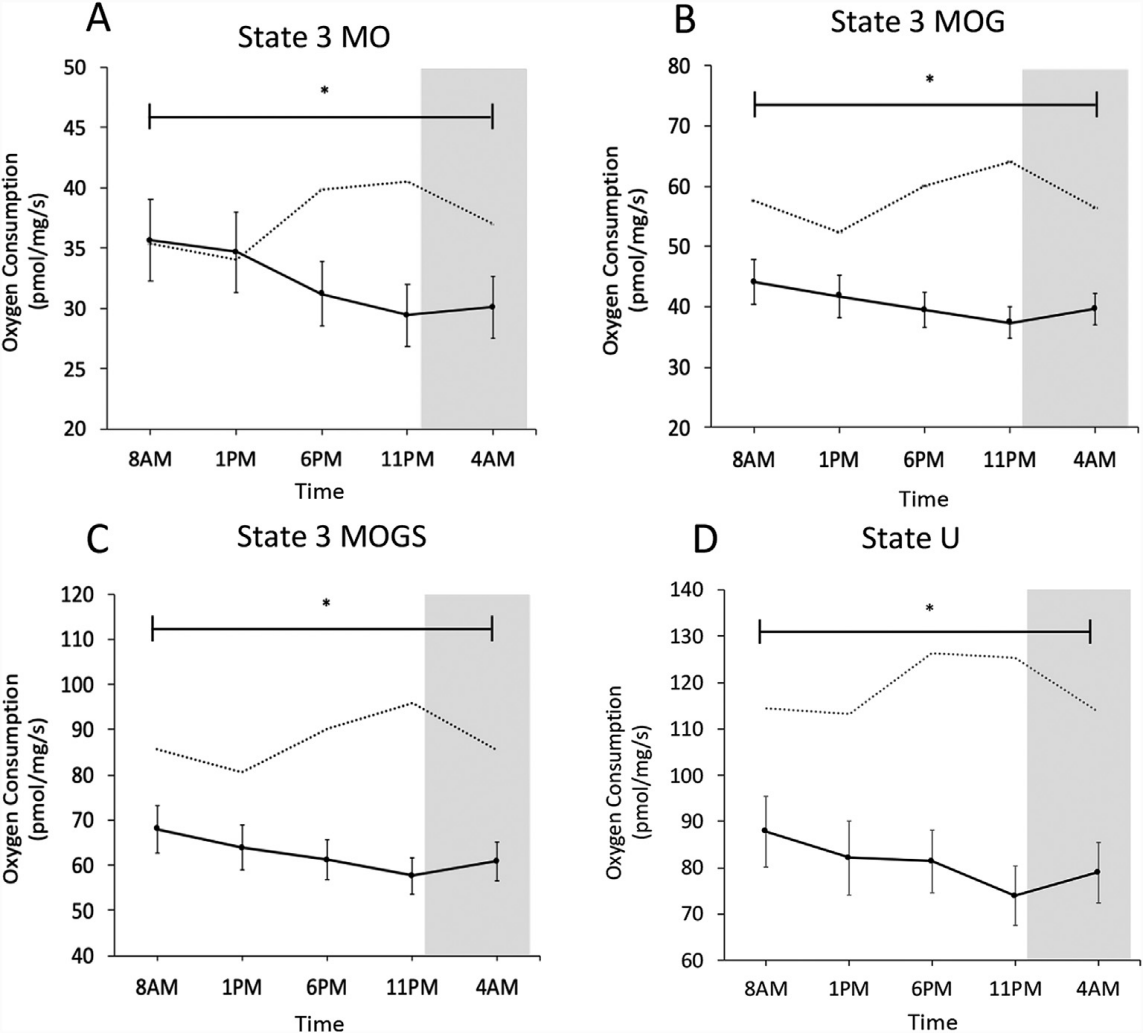
Mitochondrial oxidative capacity in skeletal muscle does not have a day-night rhythm. ADP-stimulated respiration of permeabilized muscle fibers fueled with (A) the lipid substrate octanoylcarinitine (state 3 MO); (B) addition of complex I substrates (state 3 MOG); (C) addition of substrates for parallel electron input into complex I and II (state 3 MOGS). Maximal uncoupled respiration after FCCP (State U) titration (D). For reference, we depicted the respiration states from our previous study in young, healthy, lean subjects [9] using dotted lines. M, malate; O, octanoylcarnitine; G, glutamate; S, succinate. The dark gray area represents the sleeping period (12AM–7AM). Data depicts oxygen consumption per mg wet weight per second and is shown as mean ± SEM. ∗p < 0.05 for effect of time in all states.

**Figure 2:**
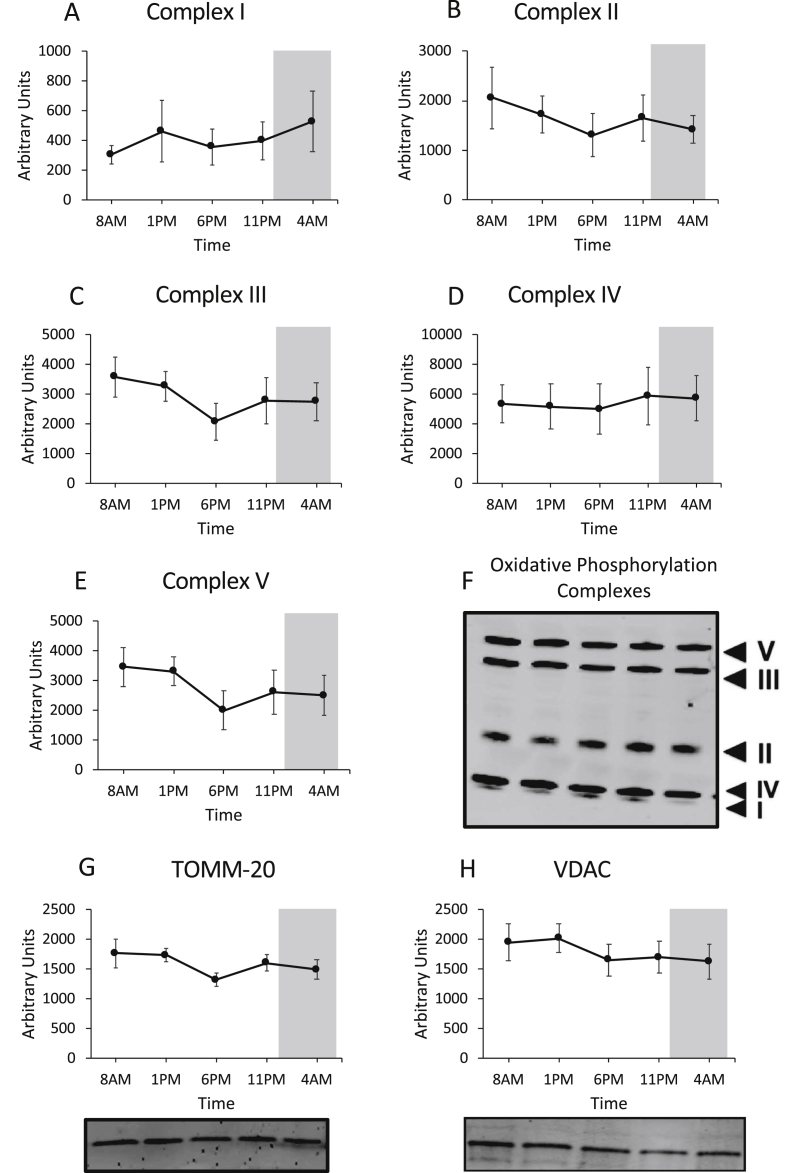
Mitochondrial respiratory chain proteins are not rhythmic. Proteins levels of oxidative phosphorylation complexes I – V (A–E). Representative western blot of one subject depicting the oxidative phosphorylation complexes of all time points (F). Protein levels of the two mitochondrial membrane proteins TOMM-20 and VDAC (G–H). Jointly, these data indicate that mitochondrial content does not possess 24-h rhythmicity. Representative western blot images are displayed below the quantification graphs. Proteins of interest were normalized to total protein content using stain-free technology. The dark gray area represents the sleeping period (12 AM–7 AM). Data is presented as mean ± SEM.

To determine potential rhythmicity in mitochondrial oxidative capacity, we performed high-resolution respirometry in freshly isolated permeabilized muscle fibers sampled at 5 timepoints over a normal day-night cycle. ADP-stimulated (state 3) mitochondrial respiration was assessed upon consecutive addition of octanoylcarnitine (O), glutamate (G), and succinate (S), with malate (M) being present as a supportive substrate (Figure 1A–C). Finally, we assessed maximal uncoupled respiration (state U) by titration of the chemical uncoupler FCCP (Figure 1D). Although we found a time effect in all mitochondrial respiration rates measured (State 3MO p = 0.005, State 3MOG p = 0.027, State 3MOGS p = 0.032, State U p = 0.043), no difference between the peak and nadir could be detected in any of the states (Bonferroni adjusted multiple testing p > 0.05). Furthermore, we performed rhythmicity analysis (JTK_Cycle) to determine whether mitochondrial respiration exhibited 24-h rhythmicity. Apart from a small but significant day-night rhythm for state 3MO respiration, with the peak at 8 AM and trough at 11 PM (35.7 ± 3.4 *vs.* 29.4 ± 2.6 pmol/mg/s, 8 AM *vs.* 11 PM, JTK_Cycle p = 0.016), mitochondrial respiration did not display day-night rhythmicity. Thus, state 3MOG and state 3MOGS respiration as well as maximal oxidative capacity (state U) did not display significant 24-h day-night rhythmicity (JTK_Cycle p > 0.05). Figure 1 further illustrates this lack of rhythmicity, as mitochondrial respiration rates merely show a flat line over time, especially when compared to our previous findings in young, healthy volunteers [9]. To investigate whether mitochondrial content is variable over the day, we measured protein levels of subunits of the oxidative phosphorylation complexes. The oxidative phosphorylation complexes I–V did not show a time effect and remained at similar levels throughout the day (Figure 2A–F). To further confirm this, we measured protein content of two mitochondrial membrane proteins, TOMM-20 and VDAC, which also showed no rhythmicity, suggesting that mitochondrial content does not change over 24 h (Figure 2G–H).

**Figure 3:**
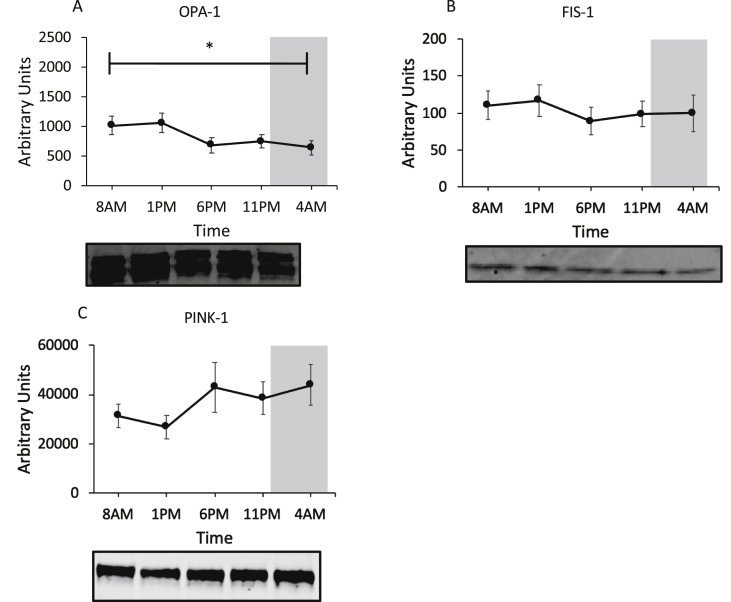
Regulators of mitochondrial dynamics. Protein levels of markers for mitochondrial fusion: OPA-1 (A); mitochondrial fission: FIS-1 (B); and mitophogy: PINK-1 (C). Representative western blots are depicted below the graphs. Marker proteins indicate no mitochondrial network remodeling over the 24-h period. Proteins of interest were normalized to total protein content using stain-free technology. The dark gray area represents the sleeping period (12 AM–7 AM). Data is presented as mean ± SEM. ∗p < 0.05 for effect of time in all states.

We previously found that markers of mitochondrial fusion and fission in lean, healthy volunteers exhibited diurnal variations, which paralleled the pattern of mitochondrial oxidative capacity [9]. We therefore investigated markers of mitochondrial fusion (OPA-1), fission (FIS-1), and mitophagy (PINK-1). Interestingly, in contrast to our previous findings, only OPA-1 levels showed a time effect (p = 0.003, Figure 3A) in this study, while FIS-1 and PINK-1 were non-rhythmic over the day. Together this could mean that the dynamic capacity of mitochondria to constantly undergo cycles of fusion and fission is impaired in these participants.

### 3.3 Whole-body substrate oxidation shows metabolic inflexibility

**Figure 4:**
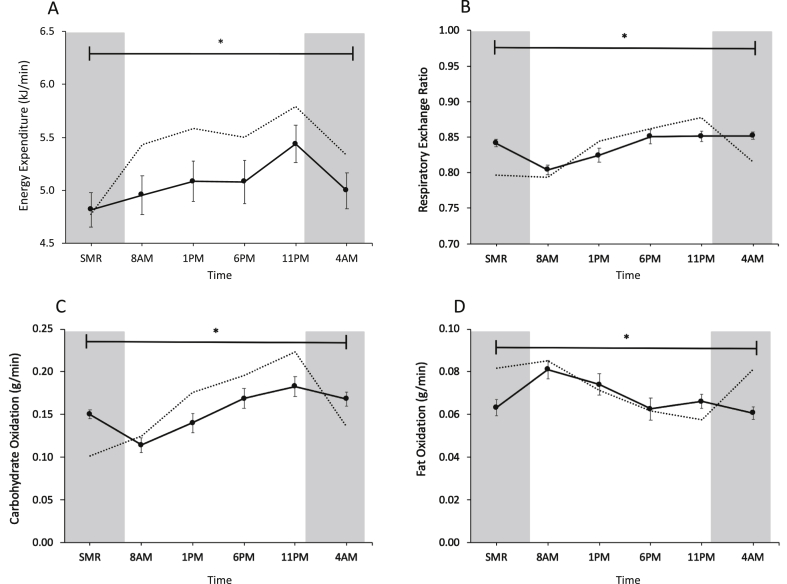
Respiratory exchange ratio shows increase in carbohydrate oxidation over the day. Whole-body resting energy expenditure (A), respiratory exchange ratio (B), carbohydrate oxidation (C), fat oxidation (D). For reference, we depicted the measurements from our earlier study in young, healthy, lean subjects [9] using dotted lines. The dark gray areas represent the sleeping periods (12 AM–7 AM) before and at the end of the test day. Data is presented as mean ± SEM. ∗p < 0.05 for effect of time.

To investigate whether whole-body energy expenditure and substrate oxidation display day-night variation, we performed indirect calorimetry in the resting state at the 5 timepoints of the mitochondrial function assessment, i.e., prior to taking each muscle biopsy. In addition, we plotted the whole-room calorimetry measurement from the first night in order to verify that measurements obtained at 4 AM after waking up were similar to an undisturbed condition while asleep (Figure 4A–D). Resting metabolic rate (RMR) showed clear differences over the day, with lowest values occurring at 8 AM and 4 AM and peak energy expenditure at 11 PM at the end of the waking period (p < 0.001 for the time effect; p = 0.001 for Bonferroni adjusted post-hoc analysis 8 AM *vs.* 11 PM; Figure 4A). RER increased over the day and plateaued in the late evening and at night, showing increased reliance on carbohydrate oxidation over the day (Figure 4B). Statistical analysis revealed a significant time effect for RER (p < 0.001 for the time effect). The mean difference between peak and trough was 0.05 ± 0.01 (0.80 ± 0.01 *vs.* 0.85 ± 0.01, 8 AM *vs.* 4 AM (p = 0.001 for Bonferroni adjusted post-hoc analysis), illustrating the relatively modest day-night variation in these older, insulin-resistant participants. For reference, the day-night variation in RER in lean, healthy volunteers in our previous study was 0.08 ± 0.02 [9]. RER remained high under fasting conditions at 4 AM even though the last meal was provided at 7 PM in the evening. As exemplified by the RER, carbohydrate oxidation increased during the day, with highest values at 11 PM (p < 0.001 for the overall time effect; p < 0.001 for Bonferroni adjusted post-hoc analysis; Figure 4C). Fat oxidation decreased accordingly, with highest fat oxidation at 8 AM (p < 0.001 for the overall time effect; p = 0.003 for Bonferroni adjusted post-hoc analysis; Figure 4D).

### 3.4 Twenty-four-hour plasma metabolites

**Figure 5:**
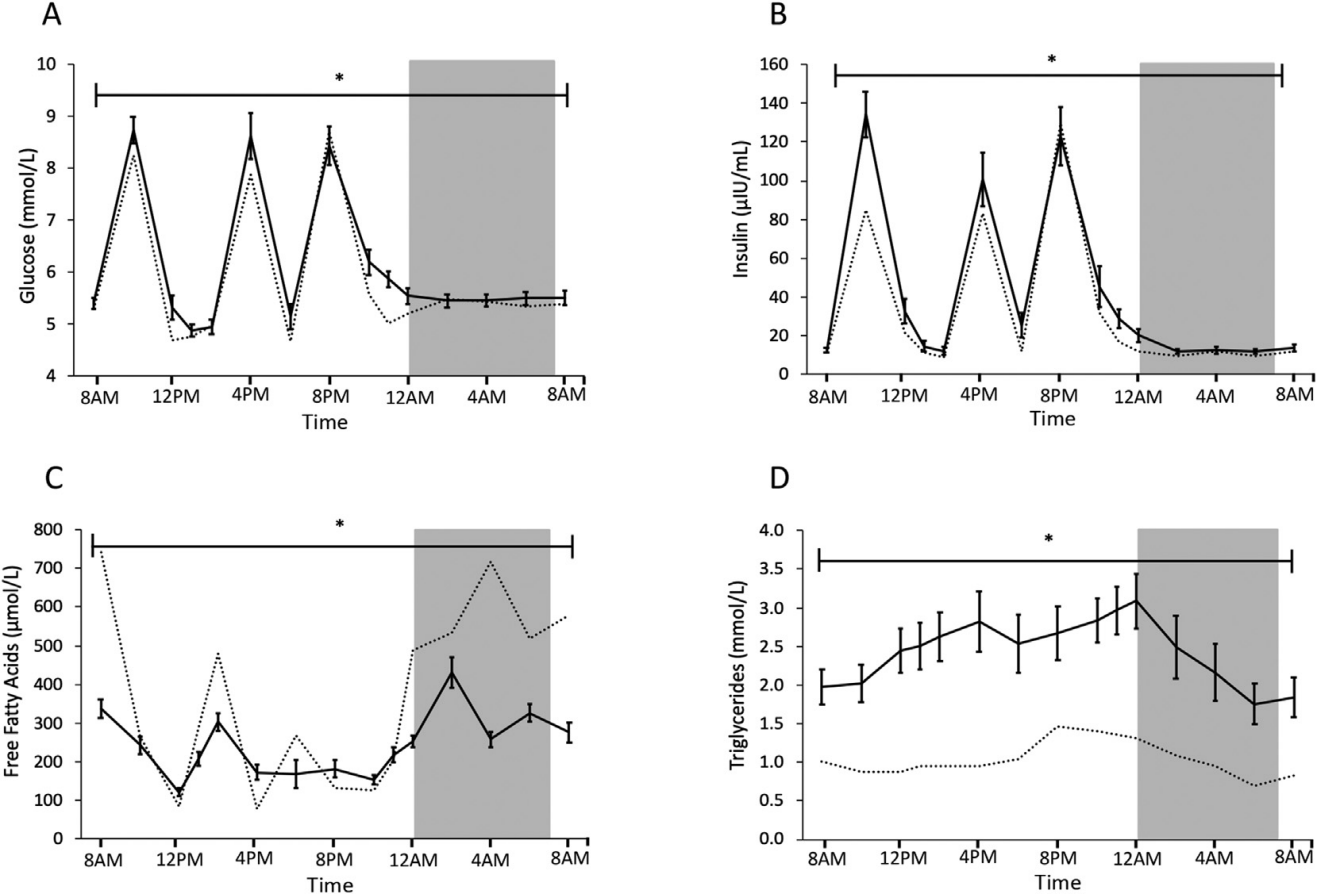
Daily variations in glucose, insulin, FFA, and triglycerides are predominantly influenced by feeding. Plasma levels of glucose (A), insulin (B), free fatty acids (C), triglycerides (D). For reference, we depicted the measurements from our earlier study in young, healthy, lean subjects [9] using dotted lines. The dark gray area represents the sleeping period (12 AM–7 AM). Data is presented as mean ± SEM. ∗p < 0.05 for effect of time.

We obtained 15 blood samples over the 24-h day-night cycle on test day 2 to determine circulating levels of metabolites. Glucose and insulin levels peaked in the postprandial periods and decreased rapidly after all meals (Figure 5A,B). Interestingly though, plasma glucose remained elevated 4 h after the dinner (11 PM), while 4 h after the breakfast (1 PM) glucose levels had decreased to the fasting level. Similar to the plasma glucose pattern, insulin levels remained high 4 h after dinner, whereas insulin levels had decreased to the fasting level 4 h after breakfast. Together, these results suggest that insulin sensitivity is lower in evening than in the morning. Free fatty acid (FFA) levels were high under fasting conditions in the morning and decreased after breakfast (9 AM) and lunch (2 PM) (Figure 5C). During the night, FFA levels increased again, with a peak at 2 AM, in agreement with the notion that during periods of fasting, FFA are released from adipose tissue to provide energy. Triglyceride (TG) levels increased over the course of the day and after each meal and kept increasing until 12 AM (midnight) – 5 h after the last meal (Figure 5D). Throughout the sleeping period (12 AM until 7 AM), TG levels decreased until they reached fasting levels the next morning.

### 3.5 Muscle clock gene analysis indicates phase shift of PER2 and CRY1

**Figure 6:**
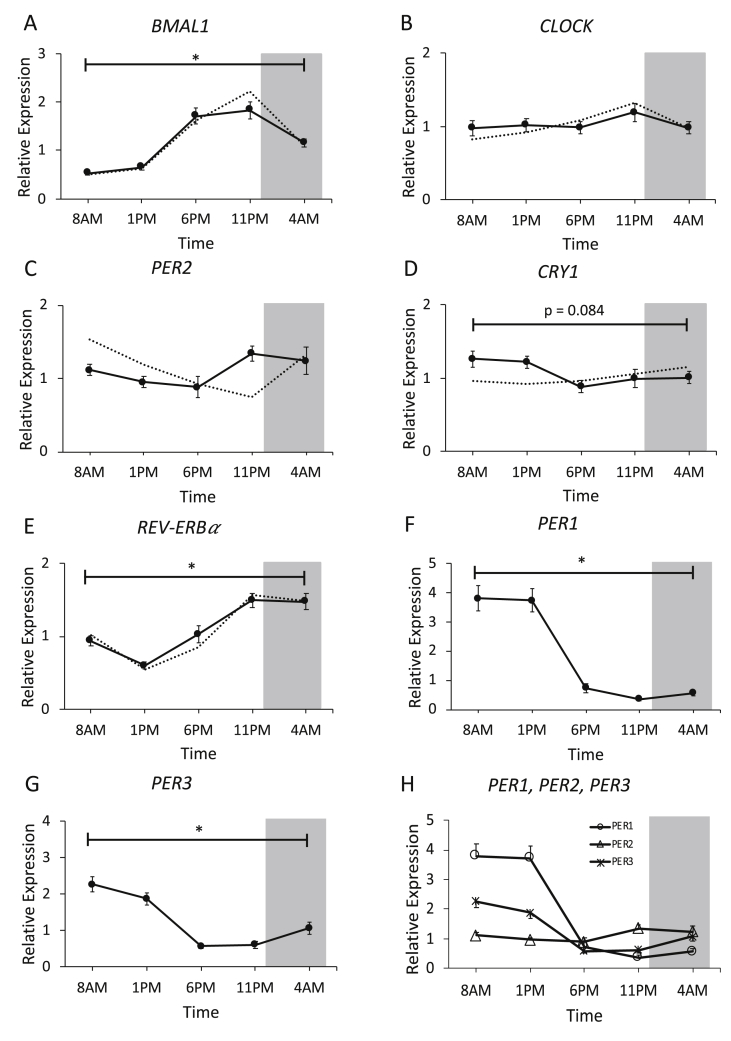
Core molecular clock gene expression in skeletal muscle. mRNA expression of *BMAL1* (A), *CLOCK* (B), *PER2* (C), *CRY1* (D), *REV-ERBα* (E), *PER1* (F), *PER3* (G), and combined expression patterns of *PER1*, *PER2*, *and PER3* in one graph (H). For reference, we depicted the mRNA expression of the representative gene from our earlier study in young, healthy, lean subjects [9] using dotted lines. Data are normalized to the geometric of 3 housekeeping genes. The dark gray area represents the sleeping period (12 AM–7 AM). Data is presented as mean ± SEM. ∗p < 0.05 for effect of time.

We and others previously reported that clock gene expression exhibited 24-h day-night rhythmicity in human skeletal muscle of lean volunteers, both when measured in human muscle biopsies and in primary human myotubes [6,9,25]. Here, we investigated whether the core clock genes display a day-night rhythm in older, metabolically compromised volunteers. To this end, we measured the positive (*BMAL1*, *CLOCK*, and *REV-ERBα*) and negative (*PER2* and *CRY1*) regulators of the muscle clock in all 5 biopsies over the day (Figure 6). Consistent with our previous findings [9], *BMAL1* and *REV-ERBα* exhibited robust oscillations, with a peak at the end of the waking period and a trough at noon (*BMAL1* p < 0.001 for the overall time effect; JTK_Cycle p < 0.001; *REV-ERBα* p < 0.001 for the overall time effect; JTK_Cycle p < 0.001; Figure 6A,E respectively). In contrast, the negative regulator *CRY1* peaked in the morning (p = 0.084 for the overall time effect, JTK_Cycle p = 0.005; Figure 6D). Even more remarkable, the expression of the negative regulator *PER2* showed that there was no day-night rhythmicity (p = 0.170 for the overall time effect; p = 0.101 for JTK_Cycle; Figure 6C). We therefore subsequently analyzed the other two PER-isoforms, *PER1* and *PER3*, and found a strong rhythmic expression pattern (*PER1*: p < 0.001 for the overall time effect; JTK_Cycle p < 0.001; *PER3* p < 0.001 for the overall time effect; JTK_Cycle p < 0.001; Figure 6F–G).

## 4 Discussion

We previously showed that skeletal muscle displays 24-h day-night rhythmicity in core molecular clock gene expression and mitochondrial oxidative capacity in young, healthy humans. This day-night rhythmicity was paralleled by day-night rhythms in whole-body energy and substrate metabolism, including a typical feeding-fasting cycle of substrate oxidation [9]. Higher age, excess body fat, and insulin resistance are all characteristic for reduced metabolic health and are typically associated with low skeletal muscle mitochondrial function [26,27] and metabolic flexibility [28]. To the best of our knowledge, this research shows for the first time in humans that skeletal muscle of older, metabolically comprised individuals is characterized by a lack of 24-h day-night rhythmicity in mitochondrial oxidative capacity and by disturbed molecular clock gene expression, paralleled by alterations in 24-h energy and substrate metabolism.

In the current study, mitochondrial oxidative capacity was determined in older, insulin-resistant or glucose intolerant participants using an identical methodology as in several of our previous studies [18,19,29], allowing the comparison of the absolute values. Mitochondrial respiration rates were clearly lower as compared to a group of young, lean participants [9], thereby confirming previous results in a comparable, older pre-diabetic population [29]. However, older, metabolically comprised participants did not show the 24-h day-night rhythmicity in mitochondrial respiration that we previously observed in young, lean, healthy volunteers (indicated with dotted lines in Figure 1 [9]). Thus, in our previous study mitochondrial oxidative capacity displayed a clear day-night rhythm with a peak in mitochondrial respiration at the end of the active period and a trough at 1 PM [9]. In the current study, however, lowest respiration occurred at 11 PM, suggesting that the peak in mitochondrial respiration that is observed in the young lean participants is completely lost. This shows that the ability to adjust mitochondrial oxidative capacity over the 24-h day-night cycle is a characteristic feature of young, lean participants, which is lost in older, overweight participants. Interestingly, this finding was paralleled by a blunted metabolic flexibility as indicated by only a marginal variation in resting RQ over the 24-h period (max variation in RQ over 24 h: 0.05 in older, overweight versus 0.08 in young, lean, see dotted line in Figure 4B). This lack of oscillation in mitochondrial oxidative capacity throughout the 24-h day-night cycle suggests that restoration of circadian rhythmicity is a potentially interesting target to improve metabolic health. The mechanisms underlying the lack of day-night rhythmicity in mitochondrial function in older, overweight volunteers cannot be deduced from the current study and requires further investigation. We found that the marker of mitochondrial fission, FIS-1, protein was not rhythmic – contrary to our previously published data – suggesting that altered mitochondrial dynamics may play a role in the absence of rhythmic respiration [30,31]. Rhythmicity of FIS1 and PINK1 on the mRNA and protein in liver is dependent on BMAL1 [30]. We therefore measured expression of these mitochondrial dynamic markers to see if the lack of rhythmicity is also present in the mRNA level. We found that *FIS-1* was borderline significant for a time effect (p = 0.052), while *PINK-1* and *BNIP-3* showed significant 24-h variation (*PINK-1* p = 0.031; *BNIP-3* p = 0.004). This discrepancy between rhythmicity on the mRNA and protein level might indicate that rhythmicity of FIS-1 and PINK-1 protein levels is affected by a post-transcriptional mechanism [32].

To investigate whether the lack of rhythmicity in mitochondrial oxidative capacity was due to a disturbed skeletal muscle circadian clock, we measured the core clock genes in skeletal muscle. The positive limb of the feedback loop (*BMAL1* and *CLOCK*) was remarkably similar to our previous study with young, lean participants. Also, the clock gene REV-ERBα was expressed almost identically in both studies. Previous research in mice showed that knockout of REV-ERBα in skeletal muscle leads to reduced mitochondrial oxidative capacity and that overexpression might increase oxidative capacity [33]. The current results do not suggest that REV-ERBα is linked to the lack of rhythmicity in mitochondrial oxidative capacity in our older, overweight participants. Contrary to the positive limb of the feedback loop, expression of the negative regulator *PER2* was not rhythmic in skeletal muscle. We also analyzed the expression of the other two isoforms of the PER gene family, *PER1* and *PER3*, which did show rhythmic expression. This finding is interesting because in mice, knockout of *PER2* leads to loss of rhythmic oxidative capacity [34]. Moreover, a recent study reported a pathway in which inflammation induced the transcription factor *NF-κB*, which directly repressed *PER2* and *CRY1*, leading to impaired rhythmicity in the liver [35]. Thus, it is possible that aging, obesity, and insulin resistance, which are associated with inflammation [36], may contribute to the observed impairment in clock gene expression. Whether the loss of rhythm in *PER2* is specific to skeletal muscle tissue remains to be established and cannot be answered from the current study. However, a recent study reported that rhythmicity of *PER2* expression in adipose tissue of both overweight T2DM patients and lean healthy controls was similar, suggesting that in adipose tissue, *PER2* expression is not altered [37].

In contrast to the lack of day-night rhythmicity in mitochondrial capacity, resting metabolic rate displayed a clear pattern over the 24-h day-night cycle with a peak in the late evening at 11 PM. This rhythm is very similar to our previous study, in which RMR displayed the same pattern, and is in agreement with another study of a 24-h measurement in resting energy expenditure [38]. Whole-body substrate oxidation changed during the day, with an increased relative carbohydrate oxidation and a lowered fat oxidation in the evening compared to the morning. The last meal was provided at 7 PM, but even several hours later (11 PM and 4 AM), the RER had not decreased. This is in contrast to our findings in young, lean participants, even though composition of the meals was similar in the 2 studies and indicates metabolic inflexibility in the older, overweight and insulin-resistant participants. A decrease in metabolic flexibility is typically observed in obesity [28], prediabetes [39], and diabetes [10], but is usually measured under fasting conditions in the morning or in the postprandial phase. In the present study, we found that metabolic inflexibility persisted throughout the entire 24-h day-night cycle and especially in the sleeping period at night, suggesting that especially at night, our participants did not enter the typical fasted state. This observation is remarkable, as volunteers were fed in energy balance, suggesting that this lack of feeding-fasting cycle is a characteristic of these older, overweight participants.

The lack of a feeding-fasting cycle is further confirmed by the higher glucose, insulin, and TG levels, as well as lower FFA levels after breakfast and lunch in comparison to the lean, young participants from our previous study, again despite that participants were fed in energy balance in both studies. Despite insulin being 60% higher 1 h after breakfast in the current study, glucose also remained elevated in comparison to the young and lean group, confirming the insulin-resistant state of the participants in the current study. Interestingly, the glucose peak after dinner was similar to the earlier study, but glucose levels only returned to fasting values at 2 AM (vs. 10 PM in young, lean participants, Figure 5A), showing that despite higher insulin levels, glucose homeostasis was not reached until 6 h after dinner. Whether this observation is only attributable to muscle insulin sensitivity or also due to blunted suppression of hepatic glucose production cannot be inferred from these data. Future studies could measure insulin signaling markers, such as phosphorylation status of AKT or AS160 in muscle biopsies taken around the clock. However, due to lack of muscle material, this was not possible in the current study. Furthermore, FFA levels – substrates that are released from adipose tissue under fasting conditions – were high in the fasted state, but had a relatively small amplitude over the 24-h period compared to the profile of young and lean participants, again suggesting that in our older, overweight population, the effect of fasting on substrate oxidation was attenuated. Elevated postprandial insulin levels may play a role herein, as insulin inhibits adipose tissue lipolysis [40]. Finally, TG levels increased during the day and only started to decline after midnight. The increase in TG during the day might be explained in part by chylomicrons that enter the circulation postprandially. Indeed, also in the previous study, TG increased clearly after dinner, which had the highest fat content of all meals (Figure 5D). However, compared to the lean, healthy volunteers, TG levels in the present study are consistently high and keep increasing until midnight, again in agreement with a prolonged fed state. An alternative mechanism underlying this observation might be increased hepatic very low density lipoprotein-triglyceride (VLDL-TG) production that is stimulated by higher postprandial insulin levels in the insulin-resistant state [41,42].

This study does not come without limitations. The lack of a control group with similar age but normal weight or insulin sensitivity prevents us from drawing conclusions as to whether aging, overweight, or insulin resistance it responsible for the lack of rhythmicity. Furthermore, we only included Caucasian men in both our current and previous study, and our findings need to be confirmed in a more diverse population.

## 5 Conclusions

We show here that in older, metabolically comprised participants, mitochondrial oxidative capacity of skeletal muscle does not exhibit a day-night rhythm. Furthermore, we report that the rhythmicity of *PER2*, a member of the internal circadian clock is affected. Additional metabolic parameters demonstrate that features of metabolic inflexibility can be observed over the full 24-h day-night cycle and that older, insulin-resistant participants do not enter a typical feeding-fasting cycle, even when fed in energy balance. Future studies are needed to explore strategies that can restore the day-night rhythm in mitochondrial oxidative capacity and substrate oxidation.

## Author contributions

J.W., N.J.C., D.v.M., M.K.C.H., B.H., J.H., and P.S. designed research; J.W., N.J.C., C.E.F., and C.A. V.d.W. performed research; J.W., C.E.F., E.M-K., J.A.J, J.H., and P.S. analyzed samples and data. J.W. and P.S. wrote the manuscript. P.S. is the guarantor of this work, and, as such, had full access to all the data in the study and takes responsibility for the integrity of the data and the accuracy of the data analysis.

## Appendix A Supplementary data

Supplemental Figure 1 Study design. Subjects stayed in a room of the metabolic research center for a total of 44 h. Meals were determined based on metabolic rate of the subjects: B, breakfast; L, lunch; D, dinner. M, measurement indicates timepoint of indirect calorimetry followed by muscle biopsy. Supplemental Figure 1

Supplemental Figure 2 Core body temperature (CBT) during day of the study protocol. Temperature (°C) was recorded every 15 s and averaged per subject into 30 min bins. The dark gray area represents the sleeping period (12 AM–7 AM). The characteristic drop in CBT beginning in the evening shows similar entrainment of the central circadian clock between subjects. Depicted is CBT mean ± SEM of n = 11 subjects. Supplemental Figure 2

Supplemental Figure 3 Gene expression of mitochondrial dynamic markers in skeletal muscle. mRNA expression of *FIS-1* (A), *PINK-1* (B), and *BNIP3* (C). Data are normalized to the geometric mean of 3 housekeeping genes. The dark gray area represents the sleeping period (12 AM–7 AM). Data is presented as mean ± SEM. ∗p < 0.05 for effect of time. Supplemental Figure 3
