# Supplementary figures and images for: Day-night rhythm of skeletal muscle metabolism is disturbed in older, metabolically compromised individuals
Source: Mol Metab. 2020 Jul 11;41:101050. doi: 10.1016/j.molmet.2020.101050 (PMC7415921; doi:10.1016/j.molmet.2020.101050)

## Supplemental Figure 1

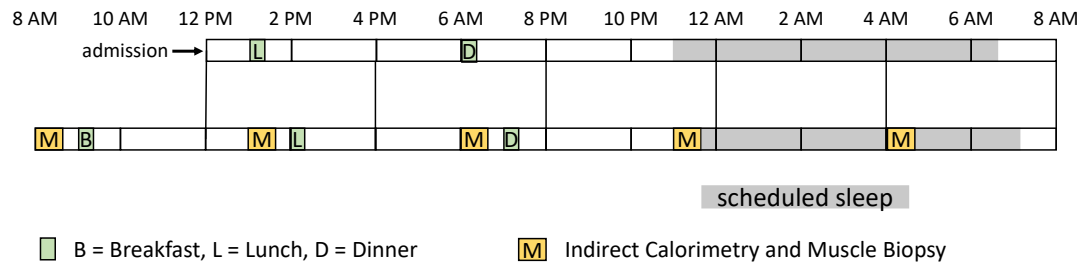

Supplement: Supplemental Figure 1 — Study design. Subjects stayed in a room of the metabolic research center for a total of 44 h. Meals were determined based on metabolic rate of the subjects: B, breakfast; L, lunch; D, dinner. M, measurement indicates timepoint of indirect calorimetry followed by muscle biopsy. [file mmc1.pdf]

Supplemental Figure 2

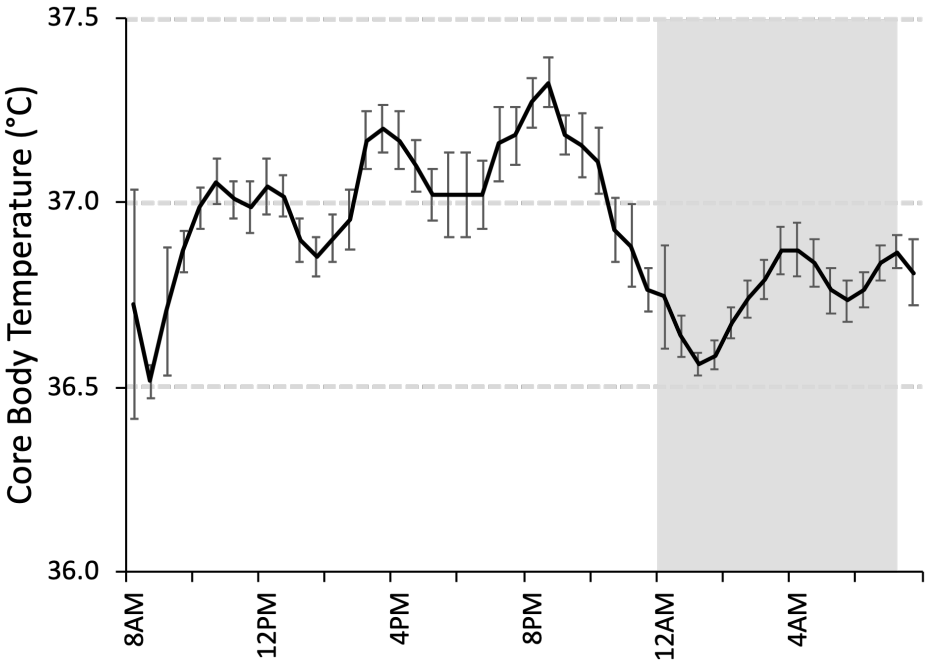

Supplement: Supplemental Figure 2 — Core body temperature (CBT) during day of the study protocol. Temperature (°C) was recorded every 15 s and averaged per subject into 30 min bins. The dark gray area represents the sleeping period (12 AM–7 AM). The characteristic drop in CBT beginning in the evening shows similar entrainment of the central circadian clock between subjects. Depicted is CBT mean ± SEM of n = 11 subjects. [file mmc2.pdf]

Supplemental Figure 3

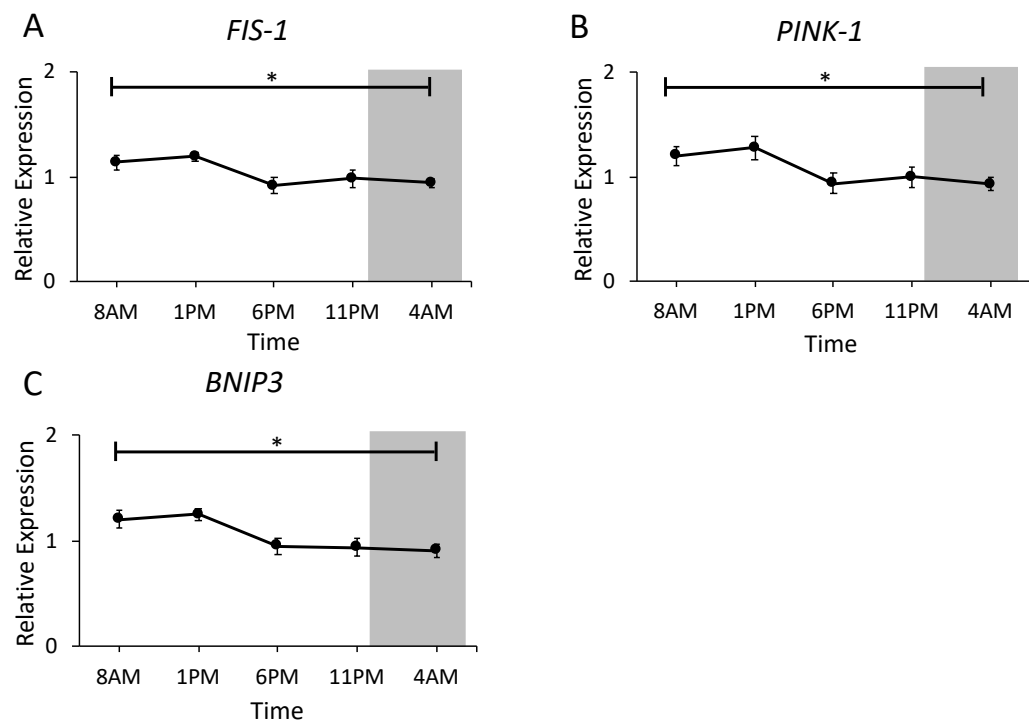

Supplement: Supplemental Figure 3 — Gene expression of mitochondrial dynamic markers in skeletal muscle. mRNA expression of FIS-1 (A), PINK-1 (B), and BNIP3 (C). Data are normalized to the geometric mean of 3 housekeeping genes. The dark gray area represents the sleeping period (12 AM–7 AM). Data is presented as mean ± SEM. ∗p < 0.05 for effect of time. [file mmc3.pdf]
